# Chronic Pain after Inguinal Hernia Repair

**DOI:** 10.1155/2014/839681

**Published:** 2014-12-15

**Authors:** Mallikarjuna Manangi, Santhosh Shivashankar, Abhishek Vijayakumar

**Affiliations:** Department of General Surgery, Victoria Hospital, Bangalore Medical College and Research Institute, Bangalore 560002, India

## Abstract

*Background*. Chronic postherniorrhaphy groin pain is defined as pain lasting >6 months after surgery, which is one of the most important complications occurring after inguinal hernia repair, which occurs with greater frequency than previously thought. *Material and Methods*. Patients undergoing elective inguinal hernioplasty in Victoria Hospital from November 2011 to May 2013 were included in the study. A total of 227 patients met the inclusion criteria and were available for followup at end of six months. Detailed preoperative, intraoperative, and postoperative details of cases were recorded according to proforma. The postoperative pain and pain at days two and seven and at end of six months were recorded on a VAS scale. *Results*. Chronic pain at six-month followup was present in 89 patients constituting 39.4% of all patients undergoing hernia repair. It was seen that 26.9% without preoperative pain developed chronic pain whereas 76.7% of patients with preoperative pain developed chronic pain. Preemptive analgesia failed to show statistical significance in development of chronic pain (*P* = 0.079). Nerve injury was present in 22 of cases; it was found that nerve injury significantly affected development of chronic pain (*P* = 0.001). On multivariate analysis, it was found that development of chronic pain following hernia surgery was dependent upon factors like preoperative pain, type of anesthesia, nerve injury, postoperative local infiltration, postoperative complication, and most importantly the early postoperative pain. *Conclusions*. In the present study, we found that chronic pain following inguinal hernia repair causes significant morbidity to patients and should not be ignored. Preemptive analgesia and operation under local anesthesia significantly affect pain. Intraoperative identification and preservation of all inguinal nerves are very important. Early diagnosis and management of chronic pain can remove suffering of the patient.

## 1. Introduction

The definition of pain devised by the International Association for the Study of Pain (IASP) is as follows: pain is an unpleasant sensory and emotional experience associated with actual or potential tissue damage, or described in terms of such damage. Chronic pain is any pain which persists beyond the normal healing period of 12 weeks. Postherniorrhaphy groin pain is defined as pain lasting >3 months after surgery, which is one of the most important complications occurring after inguinal hernia repair, and occurs with greater frequency than previously thought. A review of studies published between 1987 and 2000 showed an overall incidence of 25 percent with 10 percent of patients having pain fitting a definition of moderate or severe. Incidence of long term (≥1 year) postoperative neuralgia reported for Lichtenstein repair of inguinal hernia ranges from 6 to 29 percent [1]. Inguinodynia is the recommended generic term for chronic groin pain after hernia repair and should replace “neuralgia or mesh inguinodynia” to promote uniformity and avoid confusion in the literature.

In cases that involve workman's compensation issue, treating a postsurgical patient becomes complicated. Although most legal cases result in out-of-court settlement, worth noting is the fact that 5–7 percent of patients with postherniorrhaphy neuralgia will sue their surgeons [2]. There are various factors related to development of chronic pain following inguinal hernia repair. Preemptive analgesia, type of anesthesia, preservation of nerves, and prevention of postoperative complications are related to development of chronic pain. The most important factor in development of chronic pain is immediate postoperative pain; all measures must be taken to eliminate postoperative pain. We undertook a prospective study to analyze the factors associated with development of chronic pain following inguinal hernia repair.

## 2. Methodology

Patients undergoing elective inguinal hernioplasty in Victoria Hospital from November 2011 to May 2013 were included in the study. 227 patients were present for followup for a period of 6 months. Patients presenting with obstructed/strangulated inguinal hernia were excluded from study. Data were collected by meticulous history taking and careful examination of all patients undergoing elective inguinal hernia surgery. Since the study was performed in various surgical units of the hospital, there were slight differences in the surgical techniques. All patients underwent Lichtenstein procedure with prolene mesh of dimension 8 cm × 15 cm. The mesh was fixed with polydioxanone (PDS) sutures and skin closed in layers. Details regarding preoperative characteristics, type of anesthesia, intraoperative finding, and postoperative complication were recorded on a proforma. The pain was assessed by the Visual Analogue Scale (VAS) preoperatively and on days 1, 2, and 7 and at the end of 6 months by a questionnaire/telephonic conversation. Pain score was classified as mild VAS score 1–3, moderate VAS score 4–7, and severe VAS score >7. Patients complaining of severe pain will be called for followup for detailed examination and investigated for causes of chronic pain. The collected data was analyzed with respect to incidence and factors affecting the development of chronic pain and its management. Descriptive statistics like mean and percentage standard deviation were used to characterize patient data. The *χ* ^2^ test and Fisher's exact test were used to evaluate differences between categorical variables.

In the studies with two groups, Student's* t*-test was used to compare normally distributed continuous data and the Mann-Whitney* U* test was used to test between continuous variables that were not normally distributed. In the study with three groups, the Kruskal-Wallis ANOVA was used to analyze the continuous variables and VAS. Multivariate Cox regression analyses were performed to estimate and compare unadjusted and adjusted relative risks. A *P* value <0.05 was considered statistically significant.

## 3. Results

The present study was done at Victoria Hospital from November 2011 to May 2013. A total of 227 patients undergoing elective inguinal hernia repair satisfied the inclusion criteria and were available for followup at end of six months. The patient characteristics are summarized in Table 1. Majority of our patients were male 98.7% with mean age 49.1 years (range 18–82 years). Chronic pain at six-month followup was present in 89 patients constituting 39.4% of all patients undergoing hernia repair.  Table 2 shows the VAS scores of patient at six months following surgery. When patients were divided into groups of mild (1–3), moderate (4–7), and severe pain (>7) on basis of VAS score, it was found that majority, 30.5% (*n* = 69), had mild pain, 7.9% had moderate pain, and less than 1% had severe pain (Table 3).

Nineteen of 68 (27.9%) patients whose symptoms were of less than six-month duration developed chronic pain. Patients with symptom duration greater than six months (44%) developed chronic pain. Duration of symptoms greater than six months significantly affected development of chronic pain (*P* = 0.036) at 5% confidence interval.

It was seen that 26.9% without preoperative pain developed chronic pain whereas 76.7% of patients with preoperative pain developed chronic pain. When patients with preoperative pain were divided into two groups mild pain (<4 VAS) and moderate to severe pain (>4 VAS), it was seen that patients with significant preoperative pain had higher chances of developing chronic pain (*P* < 0.0001) at 5% confidence interval.

Only 32 patients received preemptive analgesia of which 18.7% developed chronic pain, whereas 42% of patients who did not receive preemptive analgesia developed chronic pain. Preemptive analgesia failed to show statistical significance in development of chronic pain (*P* = 0.079).

Majority (83.3%) of patients underwent hernia surgery under spinal anesthesia; this type of anesthesia had significant effect on development of chronic pain. The mean VAS scores in local group were 0.23 and 1.10 in spinal group and were statistically significant (*P* = 0.023).

Nerve identification during surgery was none in 17.2%, any one in 62.6%, and all three in 7.5% of cases on ANOVA analysis. No relation was found between nerve identification and development of chronic pain following surgery. Nerve injury was present in 22 cases and it was found that nerve injury significantly affected development of chronic pain (*P* = 0.001).

Postoperative infiltration of local anesthesia was practiced in 16.3% of cases and it was found that local infiltration at incision site significantly reduced incidence of chronic pain (*P* = 0.001).

Postoperative complications in form of hematoma, seroma, or infection were present in 8.5% of cases. It was found that postoperative complication not only increased early postoperative pain but also increased chances of development of chronic pain (*P* = 0.001).

Postoperative pain at days 1, 2, and 7 significantly affected development of chronic pain (*P* = 0.000) (Table 4).

On multivariate analysis, it was found that development of chronic pain following hernia surgery was dependent upon factors like preoperative pain, type of anesthesia, nerve injury, postoperative local infiltration, postoperative complication, and most importantly the early postoperative pain.

## 4. Discussion

Implantation of mesh is considered the “gold standard” for the treatment of inguinal hernia repair as the risk of recurrence is half compared to traditional nonmesh techniques. Ever since recurrence rates declined, attention has gradually shifted towards studying the onset of chronic pain following inguinal mesh repair, as an early study reported a staggering 63% incidence rate of chronic postoperative pain. From the mid-nineties on, somewhat lower (0–53%) incidence rates of chronic pain were published [3]. However, the need for additional research on etiology and treatment of these chronic pain syndromes following inguinal mesh repair became increasingly evident.

We studied 227 patients undergoing elective hernia surgery for development of chronic pain. The incidence of chronic pain in this study was 39.4%. Majority of patients with chronic pain had mild pain (30.5%), moderate pain was found in 7.9%, and severe incapacitating pain was less than 1%. Similarly, Poobalan [4] and coworkers found a 15–53% incidence of chronic pain in four studies with pain as the primary outcome.

### 4.1. Age

In this study, mean age of patients was 49.1 years (range 18–82 years). We found no relation between age and incidence of chronic pain. Courtney et al. [5] found that the risk of chronic pain decreased with increasing age, from 39 to 58% in patients aged less than 40 years to 14–17% in patients aged more than 65 years. The fraction of patients with severe or very severe pain was also higher in the younger group [6]. However, an overall interpretation of the data is hindered by the lack of data on physical activities, which may be different between the age groups, and consequently for their complaints.

### 4.2. Gender

In this study, only 3 patients were female; none of the patients developed chronic pain. Studies that had gender-specific data showed the highest pain incidence in women. A nationwide study of 1071 patients with a followup of 81% found 38% incidence of chronic pain in female patients compared with 28% in males (*χ* ^2^ = 3.87,  *P* < 0.05) [6]. In a study by Mori et al. [7] where 15% of 224 patients undergoing mesh hernia repair were women, three of the four patients with continuous pain were women resulting in an incidence of chronic pain of 0.5% in males versus 8.8% in females. In a retrospective study of 594 men and 56 women, 3% of males and 11% of female patients developed chronic pain [8]. In conclusion, these findings suggest that females are at a higher risk of developing chronic pain than males.

### 4.3. Preoperative Pain

In this study, it was found that 26.9% without preoperative pain developed chronic pain whereas 76.7% of patients with preoperative pain developed chronic pain. When patients with preoperative pain were divided into mild or no pain (<4 VAS) and moderate to severe pain (>4 VAS), it was found that patients with significant preoperative pain had higher chances of developing chronic pain (*P* < 0.0001). In a study by Wright et al. [9] involving 300 patients, 88% of patients that developed chronic pain had pain at the preoperative assessment, compared to 59% of patients without chronic pain (*P* < 0.001). Another study by Poobalan et al. [4] also found a significant predictive value (*P* < 0.005) between preoperative pain and chronic pain. In contrast, a large randomized study of 994 patients found no significant relation between the development of chronic and preoperative pain (*P* = 0.2) [10]. The MRC study found that 30% of patients reported no change in pain from before to after surgery but that 5% felt worse than before the herniorrhaphy [11].

The available data suggest that preoperative pain may increase the risk of developing chronic pain but more studies are required with a detailed analysis of the history and type of pain complained of in other parts of the body than the inguinal area.

### 4.4. Type of Anesthesia

Only two of the 26 cases performed under local anesthesia developed chronic pain whereas 79 of 189 cases performed under spinal anesthesia developed chronic pain. Present study results show that the use of local infiltration for inguinal hernia repair has substantial advantages over both regional and general anesthesia. Similar results were observed by a study by Nordin et al. [12]; it was also found that the longer time in theatre associated with local anesthesia was compensated for by the significantly shorter time for anesthesia, compared with regional and general anesthesia. Postoperative side effects and prolonged hospital stay after groin hernia surgery were often related to the effects of anesthesia. Local anesthesia had much better results than did its alternatives. In the regional anesthesia group patients had higher rate of micturition difficulties, some severe enough to necessitate urethral catheterization [12]. Overall cost benefit of local anesthesia over other types of anesthesia warrants its use in hernia surgery.

### 4.5. Preemptive Analgesia

Only 32 cases had preemptive analgesia in this study; it was found that preemptive analgesia did decrease incidence of chronic pain but due to limited number of cases it is difficult to recommend the same. Various studies have shown that acute pain following injury can lead to central neuronal plasticity leading to chronic pain. This can be prevented by aggressive early pain relief which might reduce chronic pain [13].

Whether techniques such as preemptive or preventive analgesia produce a clinically meaningful reduction in the intensity or duration of postsurgical pain remains unclear. Multimodal analgesic approaches that use ketamine or other N-methyl-d-aspartate receptor antagonists—gabapentin or pregabalin—COX inhibitors, steroids, and afferent neural blockade in the perioperative period have the potential to prevent central neuroplasticity. Good results have been obtained in the reduction of chronic pain after breast surgery with perioperative administration of venlafaxine, mexiletine with gabapentin, a eutectic mixture of local anesthetics (EMLA), and a combined treatment with EMLA and gabapentin [14]. All these treatments are of neuropathic rather than inflammatory pain. New studies are required to determine the value of preemptive analgesia in hernia repair and its timing and effect on development of chronic pain.

### 4.6. Nerve Sacrifice/Nerve Injury

Much controversy exists regarding which treatment to reserve for the inguinal nerves during hernia repair. Elective division of the ilioinguinal nerve has been proposed by some authors to reduce the risk of postoperative chronic pain [15].

Lichtenstein recommends always preserving the nerve to minimize the incidence of chronic pain. Some studies recommend that nerve ends be ligated to reduce the risk of chronic pain, but there were no studies on the outcome of these recommendations [16, 17]. Others have suggested that the nerves be divided or ligated only when their course in the operating field would lead to the risk of injury or if they interfere with positioning of the mesh [18].

Other studies have failed to show any relationship between the division or preservation of the ilioinguinal nerve and the risk of developing chronic pain [19]. If division of the nerve is performed, it should be as close as possible to the site where it leaves the retroperitoneum. We found nerve injury in 22 of the cases and these patients had higher incidence of chronic pain. In a study by Alfieri et al. [20], nerve injury during surgery appears to be an important factor influencing chronic pain. Chronic pain at 6 months after surgery was zero in those patients in whom all 3 nerves were identified and preserved, compared with the 40% incidence when these nerves were all divided or 4.7% when not all nerves were identified. These data would appear to suggest that if one or more nerves are not detected during surgery, it is possible that they could be inadvertently injured, entrapped, or secured, for example, if a continuous suture is introduced along the inguinal ligament or injured if the external spermatic vessels are divided to skeletonize the cord and thus generate severe pain at even some considerable time after the operation. The increased risk of developing chronic pain with the number of nerves divided can be explained by the fact that resection of the nerve has generally been performed distal to its origin, leaving the site of the injured nerve intact to continue to generate the pain signal and exposed to neuroma formation. Results from studies in which operative management of an injured nerve is reported to be responsible for severe chronic pain suggest that if the nerve identified is inadvertently divided, it is important to resect it as proximally as possible so that it would not interfere or come into contact with the mesh, thus allowing retraction of the proximal segment into the ventral muscle or retroperitoneum. The current literature is inconsistent concerning this point and opinions differ considerably.

### 4.7. Postoperative Local Infiltration

Local infiltration was followed in 37 of the cases and it was found that chronic pain developed in only 4 cases. Although studies have shown significant lower VAS scores in infiltration group, the long term effects on pain are not documented. In the study of Sinclair and colleagues [21], pain scores were reduced over the first 24 h period but not during 24–48 h. In the study by Tverskoy and colleagues [22], pain scores were reduced up to 48 h after operation.

### 4.8. Early Postoperative Pain

In the present study, it was found that early postoperative pain correlated with development of chronic pain. In a prospective study by Lau et al. [23] of 313 patients undergoing a laparoscopic repair, patients who had pain on coughing on the 6th postoperative day had a significant (*P* < 0.05) higher risk of developing chronic pain, but the method of early postoperative pain assessment was not described. In study by Heikkinen et al. [24] of 123 patients, four patients developed a chronic neuralgia type pain and had higher VAS scores on day 14 (*P* = 0.03). This finding is in agreement with a large prospective study by Callesen et al. [25] involving 466 unselected patients 1 year after surgery, where the risk of chronic pain was significantly higher in patients with a high early postoperative pain score compared to those with a lower postoperative pain score (9 versus 3%, *P* < 0.05) after 1 week. The same correlation was found in patients with severe pain after 4 weeks (24 versus 3%, *P* < 0.001). There are no studies to assess the role of specific analgesic therapies in reducing the development of a chronic pain state after inguinal herniorrhaphy. The available data suggest that the severity of early postoperative pain correlates with the risk of developing a chronic pain state. These results call for studies of the preventative effect of effective acute pain therapy.

## 5. Conclusion

In the present study, we found that chronic pain following inguinal hernia repair causes significant morbidity to patients and should not be ignored. Preemptive analgesia and operation under local anesthesia significantly affect pain. Intraoperative identification and preservation of all inguinal nerves are very important. All measures must be taken to suppress early postoperative pain and prevent complications as these lead to development of chronic pain. Early diagnosis and management of chronic pain can remove suffering of the patient.

However, the sample size and the followup period in the current study are relatively short. A larger study sample and longer followup may be needed before any further conclusion can be made.

From our study, we recommend more operations be done under local anesthesia and routine identification and preserving all nerves. Great measures must be taken to suppress early postoperative pain with multimodal analgesia. Regular followup of patient and identification of chronic pain and appropriate treatment improve patient outcome following inguinal hernia surgery.

## Figures and Tables

**Table 1:** Table showing patient characteristics.

Characteristics	Number (*n*)	Percentage (%)
Sex		
Male	224	98.70
Female	3	1.30
Preoperative pain		
Yes	56	24.70
No	171	75.30
Duration of symptoms		
<6 months	68	30.00
>6 months	159	70.00
Site		
Right	97	42.70
Left	119	52.40
Bilateral	11	4.80
Preemptive analgesia		
Yes	32	14.10
No	195	85.90
Anesthesia		
Local	26	11.50
Spinal	189	83.30
General	12	5.30
Nerve identified		
None	39	17.20
Any one	142	62.60
Two	29	12.80
All three	17	7.50
Nerve injury/cut		
None	205	90.30
Any one	17	7.50
Two	5	2.20
Three	0	0
Postoperative local infiltration		
Yes	37	16.30
No	190	83.70
Postoperative complication hematoma/infection		
Yes	19	8.40
No	208	91.60

**Table 2:** VAS score at end of 6 months.

VAS score	Frequency	Percent
0	138	60.8
1	21	9.3
2	31	13.7
3	17	7.5
4	12	5.3
5	5	2.2
6	1	.4
7	2	.9
Total	**227**	**100.0**

**Table 3:** Chronic pain incidence at six months.

Chronic pain VAS score	Number	Percentage
Mild (1–3)	69	30.5
Moderate (4–6)	18	7.9
Severe >6	2	0.9

**Table 4:** Multivariate analysis of factors for chronic pain.

Dependent variable	Type III sum of squares	df	Mean square	*F*	Significance
Duration	1.853	7	.265	1.267	.268
Analgesia	1.309	7	.187	1.564	.147
Anesthesia	3.063	7	.438	2.813	.008
Nerve identification	4.484	7	.641	1.088	.372
Nerve injury	3.086	7	.441	3.145	.003
Postop infiltration	2.120	7	.303	2.300	.028
Complications	1.307	7	.187	2.540	.016
Preop pain	170.993	7	24.428	17.068	.000
Pain day 1	156.502	7	22.357	25.530	.000
Pain day 2	208.367	7	29.767	42.166	.000
Pain day 7	230.467	7	32.924	50.482	.000
